# Hypercapnia modulates cAMP signalling and cystic fibrosis transmembrane conductance regulator‐dependent anion and fluid secretion in airway epithelia

**DOI:** 10.1113/JP271309

**Published:** 2015-12-20

**Authors:** Mark J. Turner, Vinciane Saint‐Criq, Waseema Patel, Salam H. Ibrahim, Bernard Verdon, Christopher Ward, James P. Garnett, Robert Tarran, Martin J. Cann, Michael A. Gray

**Affiliations:** ^1^ Institute for Cell & Molecular Biosciences, The Medical SchoolNewcastle UniversityFramlington PlaceNewcastle upon TyneNE2 4HHUK; ^2^ Institute for Cellular Medicine, The Medical SchoolNewcastle UniversityFramlington PlaceNewcastle upon TyneNE2 4HHUK; ^3^ Marsico Lung InstituteUniversity of North CarolinaChapel HillNC27599USA; ^4^ School of Biological and Biomedical SciencesDurham UniversitySouth RoadDurhamDH1 3LEUK; ^5^ Department of Physiology, McIntyre Medical Sciences BuildingMcGill University3655 Promenade Sir William OslerMontrealQuebecCanadaH3G 1Y6

## Abstract

**Key points:**

- Raised arterial blood CO_2_ (hypercapnia) is a feature of many lung diseases.
- CO_2_ has been shown to act as a cell signalling molecule in human cells, notably by influencing the levels of cell signalling second messengers: cAMP and Ca^2+^.
- Hypercapnia reduced cAMP‐stimulated cystic fibrosis transmembrane conductance regulator‐dependent anion and fluid transport in Calu‐3 cells and primary human airway epithelia but did not affect cAMP‐regulated HCO_3_^−^ transport *via* pendrin or Na^+^/HCO_3_^−^ cotransporters.
- These results further support the role of CO_2_ as a cell signalling molecule and suggests CO_2_‐induced reductions in airway anion and fluid transport may impair innate defence mechanisms of the lungs.

**Abstract:**

Hypercapnia is clinically defined as an arterial blood partial pressure of CO_2_ of above 40 mmHg and is a feature of chronic lung disease. In previous studies we have demonstrated that hypercapnia modulates agonist‐stimulated cAMP levels through effects on transmembrane adenylyl cyclase activity. In the airways, cAMP is known to regulate cystic fibrosis transmembrane conductance regulator (CFTR)‐mediated anion and fluid secretion, which contributes to airway surface liquid homeostasis. The aim of the current work was to investigate if hypercapnia could modulate cAMP‐regulated ion and fluid transport in human airway epithelial cells. We found that acute exposure to hypercapnia significantly reduced forskolin‐stimulated elevations in intracellular cAMP as well as both adenosine‐ and forskolin‐stimulated increases in CFTR‐dependent transepithelial short‐circuit current, in polarised cultures of Calu‐3 human airway cells. This CO_2_‐induced reduction in anion secretion was not due to a decrease in HCO_3_^−^ transport given that neither a change in CFTR‐dependent HCO_3_^−^ efflux nor Na^+^/HCO_3_^−^ cotransporter‐dependent HCO_3_^−^ influx were CO_2_‐sensitive. Hypercapnia also reduced the volume of forskolin‐stimulated fluid secretion over 24 h, yet had no effect on the HCO_3_^−^ content of the secreted fluid. Our data reveal that hypercapnia reduces CFTR‐dependent, electrogenic Cl^−^ and fluid secretion, but not CFTR‐dependent HCO_3_^−^ secretion, which highlights a differential sensitivity of Cl^−^ and HCO_3_^−^ transporters to raised CO_2_ in Calu‐3 cells. Hypercapnia also reduced forskolin‐stimulated CFTR‐dependent anion secretion in primary human airway epithelia. Based on current models of airways biology, a reduction in fluid secretion, associated with hypercapnia, would be predicted to have important consequences for airways hydration and the innate defence mechanisms of the lungs.

## Key points

- Raised arterial blood CO_2_ (hypercapnia) is a feature of many lung diseases.
- CO_2_ has been shown to act as a cell signalling molecule in human cells, notably by influencing the levels of cell signalling second messengers: cAMP and Ca^2+^.
- Hypercapnia reduced cAMP‐stimulated cystic fibrosis transmembrane conductance regulator‐dependent anion and fluid transport in Calu‐3 cells and primary human airway epithelia but did not affect cAMP‐regulated HCO_3_^−^ transport *via* pendrin or Na^+^/HCO_3_^−^ cotransporters.
- These results further support the role of CO_2_ as a cell signalling molecule and suggests CO_2_‐induced reductions in airway anion and fluid transport may impair innate defence mechanisms of the lungs.

**Abbreviations**
CF: cystic fibrosis
CFTR: cystic fibrosis transmembrane conductance regulator
*I*_sc_: short circuit current
NBC: Na^+^/HCO_3_^−^ cotransporter
NHE: Na^+^/H^+^ exchanger
pH_i_: intracellular pH
pH_e_: extracellular pH
PKA: protein kinase A
sAC: soluble adenylyl cyclase
tmAC: transmembrane adenylyl cyclase
*V*_te_: transepithelial voltage

## Introduction

Carbon dioxide constitutes 0.04% by volume of the Earth's atmosphere (van der Laan‐Luijkx *et al*. 2013) and has major roles in plant, prokaryote and animal biology (Cummins *et al*. 2014). In plants, CO_2_ is used to synthesise sugars during photosynthesis whilst in animals, although CO_2_ is a waste product of cellular respiration, it also has an important roles in maintaining plasma pH *via* its buffering effect on HCO_3_^−^ (Marques *et al*. 2003) as well as stimulation of peripheral and central chemoreceptors to regulate ventilation (Somers *et al*. 1989; Guyenet *et al*. 2010). Elevated CO_2_ in arterial blood (hypercapnia) is associated with lung disease in humans (Lourenco & Miranda, 1968; Prin *et al*. 2002), yet the effects of hypercapnia in human physiology are not fully understood. In mammals, recent studies have provided strong evidence that CO_2_ can act as a *bona fide* cell signalling molecule, and that changes in CO_2_ alter the activity of a variety of membrane transporters, including connexin 26 (Huckstepp *et al*. 2010 *a*,*b*; Meigh *et al*. 2013), the epithelial Na^+^/HCO_3_^−^ cotransporter (NBC) (Adijanto *et al*. 2009), inwardly rectifying K^+^ channels (Huckstepp & Dale, 2011) and the Na^+^/K^+^‐ATPase (Briva *et al*. 2007; Vadasz *et al*. 2008). The action of CO_2_ on membrane transporters has been shown to involve different mechanisms. For instance, CO_2_‐dependent downregulation of Na^+^/K^+^‐ATPase activity specifically involves the endocytosis of the α subunit of the Na^+^/K^+^‐ATPase, demonstrating that CO_2_ can alter surface expression of ion transporters (Briva *et al*. 2007). Alternatively, CO_2_ directly modulates connexin 26 *via* carbamylation, a post‐translational modification whereby a covalent bond forms between the carbon in CO_2_ and a primary amine group of the target protein (Meigh *et al*. 2013). In addition, CO_2_ also has reported effects on key cell second messengers involved in membrane transporter regulation, specifically cAMP and Ca^2+^ (Cann *et al*. 2003; Cann, 2004). cAMP is synthesised from ATP, a reaction catalysed by adenylyl cyclase, of which there exists both membrane‐bound transmembrane adenylyl cyclase (tmAC) and the soluble adenylyl cyclase (sAC) in mammals (Buck *et al*. 1999). Our laboratory has previously shown that the activity of a recombinant, catalytically active mammalian tmAC, expressed in HEK 293T cells, was significantly higher in cells exposed to 5% CO_2_ compared to those exposed to 0.03% CO_2_, demonstrating that tmAC is sensitive to changes in CO_2_ (Townsend *et al*. 2009). This study also showed that tmAC was sensitive to CO_2_ but not HCO_3_^−^ *in vivo* and *in vitro*, supporting previous findings that first proposed tmAC activity was only sensitive to CO_2_ and not inorganic carbon *per se* (Hammer *et al*. 2006). More recently, we have shown that incubating OK cells (a model of human proximal tubule cells) in 10% CO_2_ caused a significant reduction in both forskolin and parathyroid hormone‐stimulated increases in intracellular cAMP ([cAMP]_i_) compared to levels measured under normocapnic conditions of 5% CO_2_ (Cook *et al*. 2012). The decrease in cAMP correlated with an enhanced activity of the Na^+^/H^+^ exchanger (NHE) 3, a transporter known to be negatively regulated by cAMP/protein kinase A (PKA), thus providing evidence that hypercapnia was able to modulate cAMP‐regulated transporters in human epithelial cells. This work further showed that the effect of raised CO_2_ on cAMP was dependent on an IP_3_‐dependent release of Ca^2+^ which, in turn, led to an inhibition in tmAC activity, thereby demonstrating that CO_2_ affected Ca^2+^ as well as cAMP signalling. These data supported earlier studies that demonstrated CO_2_ modulated Ca^2+^ signalling in other mammalian and human cells (Nishio *et al*. 2001; Bouyer *et al*. 2003; Briva *et al*. 2011).

In the airways, cAMP plays a major role in regulating the volume and composition of the airway surface liquid (ASL). In the upper airways, ASL secretion occurs predominantly from serous cells of the submucosal glands (SMGs). Studies on intact SMG secretions as well as SMG‐derived secretory cell lines, such as Calu‐3, have found that elevations in intracellular cAMP stimulate cystic fibrosis transmembrane conductance regulator (CFTR)‐dependent Cl^−^, HCO_3_^−^ and fluid transport (Lee *et al*. 1998; Devor *et al*. 1999; Joo *et al*. 2002; Krouse *et al*. 2004; Ballard *et al*. 2006; Ianowski *et al*. 2007; Lee & Foskett, 2010; Garnett *et al*. 2011; Huang *et al*. 2012; Shan *et al*. 2012). Efficient anion secretion in the airways is paramount to maintain ASL hydration and pH, as well as efficient mucus secretion and expansion (Garcia *et al*. 2009; Chen *et al*. 2010; Gustafsson *et al*. 2012; Ridley *et al*. 2014). Loss of functional expression of CFTR at the apical membrane of HCO_3_^−^‐secreting epithelia underlies the hereditary disease cystic fibrosis (CF) and airways dehydration and impaired ASL alkalinisation have been reported in CF airways (Coakley *et al*. 2003; Song *et al*. 2006; Boucher, 2007) consistent with a key role for CFTR in mediating airway HCO_3_^−^ secretion. Furthermore, it has been shown that the acidic ASL found in CF pigs compromises the ability to kill airway pathogens (Pezzulo *et al*. 2012) and provides a plausible explanation as to why CF patients are susceptible to airway bacterial colonisation.

Given the previously reported findings from our laboratory that hypercapnia modulated cAMP signalling in renal epithelial cells (Cook *et al*. 2012), we hypothesised that hypercapnia would also affect airway epithelial cell function. Our results show that hypercapnia reduced cAMP levels in Calu‐3 cells and this correlated with a drop in cAMP‐dependent anion secretion. The reduction in anion secretion appeared primarily due to a reduction in Cl^−^ transport, given that both CFTR‐dependent HCO_3_^−^ efflux *via* pendrin, and NBC‐dependent HCO_3_^−^ import were unaffected by hypercapnia. Furthermore, hypercapnia also reduced the volume of cAMP‐stimulated fluid secretion without affecting the HCO_3_^−^ content of the fluid, implying that Cl^−^ secretion and HCO_3_^−^ secretion have differential sensitivities to hypercapnia. Hypercapnia also reduced cAMP‐stimulated anion secretion in primary human bronchial epithelial layers, indicating this effect of CO_2_ would be predicted to occur *in vivo*. Our results therefore demonstrate that CO_2_ acts as a signalling molecule in human airway epithelia to downregulate anion and fluid secretion.

## Methods

### Calu‐3 cell culture

The human serous cell line, Calu‐3 (Shen *et al*. 1994), was grown in Eagle's minimum essential medium supplemented with 10% (v/v) fetal calf serum, 1% (v/v) non‐essential amino acids, 2 mm l‐glutamine, 100 U ml^−1^ penicillin and 100 μg ml^−1^ streptomycin. Cells were incubated at 37°C in humidified air containing 5% (v/v) CO_2_ and were used between passage 20 and 50. Unless otherwise stated, 250,000 cells were seeded onto either 12 mm Costar Transwells or 12 mm Snapwells, 0.4 μm pore size, polyester membrane inserts, and grown under submerged conditions with 500 μl growth media applied to the apical compartment of membrane inserts. Transepithelial electrical resistance (TEER) was routinely measured using an epithelial voltohmmeter (WPI, Hitchin, UK) and cells generally reached a confluent monolayer, with a TEER of above 600 Ω cm^−2^ after 6 days of growth on Transwell inserts. Experiments were performed 9–13 days after seeding.

### Primary human bronchial epithelial cell culture

Ethical approval was granted for this work from Newcastle and North Tyneside 2 [Min Ref: 2001/179]. Differentiated primary bronchial epithelial cells were derived from bronchial brushings taken from lung transplant recipients during surveillance bronchoscopy as previously described (Forrest *et al*. 2005). These were grown in a CO_2_ incubator (37°C; 5% CO_2_) to 90% confluence using bronchial epithelial growth medium with supplements (BEGM, Lonza, Tewkesbury, UK) in T25 flasks pre‐coated with 32 μg ml^−1^ collagen. Cells were passaged using a standard trypsin/EDTA technique and cryopreserved for future use. After reconstitution, cells were again expanded to near confluence in T25 flasks, before being seeded onto collagen‐coated 12 mm Costar Snapwells at a density of 100,000 cells per membrane in 0.5 ml BEGM, with 2 ml of this medium applied to the basal chamber. Confluence was reached after 72 h, at which point the cell culture was taken to the air–liquid interface (ALI). Here, the medium above the cells was removed completely, and the cells were subsequently fed only from the basal chamber with an ALI medium as described by Fulcher *et al*. (2005). Ciliogenesis was first observed at 14 days at the ALI, and short‐circuit current measurements were performed 30–35 days after growth at the ALI.

### Short‐circuit current measurements

Cells were grown on Snapwell inserts and mounted into a Ussing chamber in which each chamber was connected to a calomel voltage sensing electrode and an AgCl_2_ current sensing electrode by 3 m KCl salt bridges containing 3% (w/v) agar. Cells were bathed in 7.5 ml Krebs solution and continually gassed with either 5% (v/v) CO_2_/95% (v/v) O_2_ for control conditions or 10% (v/v) CO_2_/90% (v/v) O_2_ to induce hypercapnia. To measure the short circuit current (*I*_sc_), cells were clamped at 0 mV using a DVC‐1000 Voltage/Current Clamp (WPI) and a Powerlab 1200 feedback amplifier (AD Instruments, Oxford, UK) injected the appropriate current to clamp transepithelial voltage (*V*_te_) to 0 mV, which was recorded as the *I*_sc_ using Scope 3 software (AD Instruments). To monitor transepithelial resistance (*R*_te_), a 2 s, 10 mV pulse was applied every 30 s.

### Intracellular pH measurements

Calu‐3 cells were grown on Transwell inserts and loaded with the pH‐sensitive, fluorescent dye BCECF‐AM (10 μm) for 1 h in a NaHEPES buffered solution at 37°C. Cells were mounted on to the stage of a Nikon fluor inverted microscope and perfused with a modified Krebs solution gassed with either 5% (v/v) CO_2_/95% (v/v) O_2_ or 10% (v/v) CO_2_/90% (v/v) O_2_. Solutions were perfused across the apical and basolateral membranes at 37°C at a speed of 3 ml min^−1^ (apical) and 6 ml min^−1^ (basolateral). Intracellular pH (pH_i_) was measured using a Life Sciences Microfluorimeter System in which cells were alternately excited at 490 and 440 nm wavelengths every 1.024 s with emitted light collected at 510 nm. The ratio of 490 to 440 nm emission was recorded using PhoCal 1.6 b software and calibrated to pH_i_ using the high K^+^/nigericin technique (Hegyi *et al*. 2003) in which cells were exposed to high K^+^ solutions containing 10 μm nigericin, set to a desired pH, ranging from 6.6 to 8.4. Total buffering capacity (β_tot_) was calculated by addition of the intrinsic buffering capacity (β_i_) to the buffering capacity of the CO_2_–HCO_3_^−^ buffer system (βHCO_3_^−^) in which β_i_ was calculated using the NH_4_^+^ technique as described by Roos and Boron (1981). For analysis of pH_i_ measurements, ΔpH_i_ was determined by calculating the mean pH_i_ over 60 s resulting from treatment. The rate of pH_i_ change (ΔpH_i_/Δ*t*) was determined by performing a linear regression over a period of at least 30 s which was converted to a transmembrane HCO_3_^−^ flux [–*J*(*B*)] by multiplying ΔpH_i_/Δ*t* by β_tot_.

### Radiolabelled cAMP assay

Calu‐3 cells were cultured in Corning 12‐well plates at an initial seeding density of 3 × 10^5^ cells per well and used at approximately 80% confluency. Cells were loaded with 2 μCi ml^−1^ [^3^H]‐adenine and incubated for 2 h at 37°C in humidified air containing 5% (v/v) CO_2_. Cells were then washed twice with PBS and incubated for a further 30 min at 37°C in humidified air containing 5% (v/v) CO_2_/95% (v/v) O_2_ (normocapnic controls) or 10% (v/v) CO_2_/90% (v/v) O_2_ (hypercapnia). Incubation was performed in growth medium containing 1 mm 3‐isobutyl‐1‐methylxanthine (IBMX) that had been pregassed with the appropriate CO_2_ concentration and titrated to pH 7.4 using 1 m NaOH. Forskolin (5 μm) was then added to the cells for 10 min before the assay was ended by removal of media and lysis of cells by adding 5% (w/v) trichloroacetic acid containing 1 mm ATP and 1 mm cAMP for 1 h at 4°C. cAMP levels in lysates were measured by the twin column chromatography procedure described by Johnson *et al*. (1994).

### Cell surface biotinylation

Calu‐3 cells were grown on Transwell inserts and washed three times with PBS. Cells were then incubated at 37°C in humidified air containing 5% (v/v) CO_2_ (control) or 10% (v/v) CO_2_ (hypercapnia) in pregassed high Cl^−^ Krebs solution for 20 min. The solution was removed and cells were incubated for 30 min at 4°C in PBS++ (PBS containing 0.1 mm Ca^2+^ and 1 mm Mg^2+^; pH 8.0) with 0.5 mg ml^−1^ EZ‐Link Sulfo‐NHS‐Biotin (Thermo Scientific, Waltham, MA, USA) added to the apical membrane. Biotinylation was stopped by removal of the apical solution and addition of ice cold PBS++. Cells were then lysed using RIPA buffer containing 150 mm NaCl, 20 mm Tris, 1% Triton‐X‐100, 0.1% SDS and 0.08% sodium deoxycholate (pH8.0) with one protease inhibitor cocktail tablet (Roche Applied Sciences, Penzberg, Germany) added to 50 ml RIPA buffer. The lysate was collected and centrifuged for 15 min at 16,200 × g at 4°C and the protein concentration of the supernatant was assessed using the BCA protein assay kit (Pierce Biotechnology, Rockford, IL, USA). One hundred micrograms of protein was taken to be used for analysis of whole cell protein expression. Streptavidin agarose beads (Novagen, Billerica, MA, USA) that had been equilibrated with PBS++ and RIPA buffer were added to the remaining protein at 1 μl beads/20 μg protein and incubated overnight at 4°C with continuous inversion of samples to ensure thorough mixing. These samples were then centrifuged and washed five times with RIPA buffer and heated to 65°C for 5 min. Protein expression was then detected by Western blot.

### Western blot

SDS‐PAGE using 7% gels was performed on all samples at 120 V for 2 h. Samples were then transferred to a nitrocellulose membrane at 400 mA for 90 min at 4°C. The membrane was blocked for 1 h in blocking buffer consisting of Tris‐buffered saline (TBS) + 0.1% Tween 20 (TTBS) containing 5% dried skimmed milk powder (Compliments) before primary mouse anti‐CFTR monoclonal antibody 23C5 (1:200 dilution in TBS) and mouse anti‐α tubulin antibody (1:1000 dilution in TBS) were added overnight at 4°C. The membrane was then washed using TTBS before a goat anti‐mouse antibody labelled with HRP was added at 1:5000 dilution in TBS for 1 h. Any unbound secondary antibody was then washed off with TTBS. To detect any HRP activity, equal volumes of the enhanced chemiluminescent substrates Enhanced Luminol Reagent and the Oxidizing Reagent (Thermo Scientific) were added to the blot for 10 min before the blot was exposed to Kodak Scientific Imaging film for 30 s. The film was developed and the band intensity was analysed using ImageJ software.

### Fluid secretion assays

Calu‐3 cells were grown on Transwell inserts and washed three times with PBS to remove any mucus that may have accumulated over time. Extra care was taken when removing the PBS to ensure no residual fluid remained in the Transwell at the end of the washes. Solutions were then added to the cells (1 ml basolaterally, 200 μl apically) and cells were incubated at 37°C in humidified air containing 5% (v/v) CO_2_ (control) or 10% (v/v) CO_2_ (hypercapnia) for 24 h (Garnett *et al*. 2011). The apical fluid was then removed and its volume was measured. First, 180 μl was removed and then the rest of the fluid was removed 1 μl at a time to ensure high accuracy. Samples were collected in an Eppendorf tube and after a full equilibration in either 5 or 10 % CO_2_, pH was assessed using a MiniTrode lab pH electrode (Hamilton, Reno, NV, USA). This enabled the HCO_3_^−^ concentration of the secreted fluid to be calculated using the Henderson–Hasselbalch equation, where;  pH =pKa+ lo g10([ HCO 3−]/(0.03×PCO2)) where pK_a_ = 6.1 (the negative log of the carbonic acid dissociation constant).

### Periodic acid‐Schiffs (PAS) assay

Given that it has been reported that Calu‐3 cells secrete mucins, notably MUC5AC (Kreda *et al*. 2007, 2010), the PAS assay was used to detect the glycoprotein content of the secreted fluid as an indicator of secreted mucin. To generate a standard curve, pig mucin (a gift from Prof. Jeff Pearson, Newcastle University) was diluted to 100, 50, 20, 10, 5, 2 and 1 μg ml^−1^ and 100 μl of standards was added to a 96‐well plate in duplicate. Then, 100 μl of sample was made to 1 ml by addition of deionised water and 100 μl was added to wells in duplicate. One hundred microlitres of a periodic acid/acetic acid mix (made from 10 μl periodic acid added to 7% acetic acid) was added to all standards and samples and the plate were incubated for 60 min at 37°C. In total, 100 μl of 1.6% sodium metabisulphate solution in Schiff's reagent was added to all standards and samples. The plate was then incubated at room temperature for 30 min before absorbance was read at 550 nm using an ELx808 Absorbance Microplate Reader (BioTek, Winooski, VT, USA). Absorbance was then converted to mucin concentration using the standard curve.

### Solutions and reagents

All reagents were purchased from Sigma Aldrich (Poole, UK) apart from forskolin and ouabain (R & D Systems, Abingdon, UK), BCECF‐AM (Invitrogen, Paisley, UK) and GlyH‐101 and CFTR_inh_ 172 (Calbiochem, Watford, UK). All gas cylinders were purchased from BOC (Guildford, UK) and consisted of the following mixtures: 5% CO_2_/95% O_2_ and 10% CO_2_/90% O_2._ NaHEPES solution consisted of (in mm) 130 NaCl, 5 KCl, 1 CaCl_2_, 1 MgCl_2_, 10 NaHEPES and 10 d‐glucose, pH 7.4 at 37°C. High Cl^−^ Krebs solution consisted of (in mm) 25 NaHCO_3_, 115 NaCl, 5 KCl, 1 CaCl_2_ 1 MgCl_2_ and 10 d‐glucose (pH 7.4). For high Cl^−^, Na^+^‐free solutions, NaHCO_3_ was replaced with choline bicarbonate and NaCl was replaced with *N*‐methyl‐d‐glucamine (NMDG)‐Cl. Zero Cl^−^ Krebs solution consisted of (in mm) 25 NaHCO_3_, 115 Na‐gluconate, 2.5 K_2_SO_4_, 1 Ca‐gluconate, 1 Mg‐gluconate and 10 d‐glucose. pH_i_ calibration solutions consisted of (in mm) 5 NaCl, 130 KCl, 1 CaCl_2_, 1 MgCl_2_, 10 d‐glucose, 10 HEPES (for solutions set at pH 7.6 or below) or 10 Tris (for solutions set at pH 7.8 or above) as well as 10 μm nigericin. Solutions were set to the desired pH by using 1 m HCl or 1 m NaOH. Solutions used to determine intracellular buffering capacity consisted of (in mm) 4.5 KCl, 1 MgCl_2_, 2 CaCl_2_, 5 BaCl, 10 Hepes, 10 d‐glucose as well as varying concentrations of NH_4_Cl/NMDG‐Cl, ranging from 0 NH_4_Cl/145 NMDG‐Cl to 30 NH_4_Cl/115 NMDG‐Cl. All solutions were titrated to pH 7.4 at 37°C using 1 m CsOH.

### Statistical analysis

Statistical analysis was performed using GraphPad Prism 4 software. Results are expressed as mean ± SEM of *n* observations. Student's *t* test, one‐way ANOVA (with Tukey's multiple comparison post‐test) or two‐way ANOVA (with Bonferroni post‐test) were carried out where applicable to determine statistical significance between measurements. A *P* value of < 0.05 was considered statistically significant.

## Results

### Acute hypercapnia attenuates forskolin‐stimulated cAMP levels in Calu‐3 cells independent of changes in pH_i_

**Figure 1:**
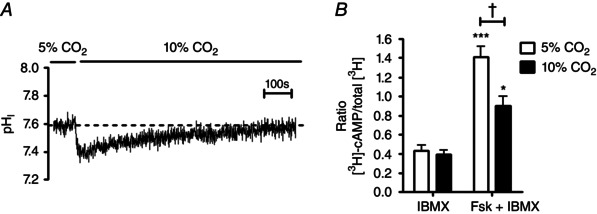
**Acute hypercapnia attenuates forskolin‐stimulated cAMP levels in Calu‐3 cells independent of changes in intracellular pH** *A*, the effect of hypercapnia (10% CO_2_) on the pH_i_ of Calu‐3 cells; cells recovered pH_i_ from CO_2_‐induced acidosis after ∼20 min. *B*, the effect of acute hypercapnia on intracellular cAMP in which cells were incubated for 20 min in either 5% CO_2_ (v/v) in air or 10% CO_2_ (v/v) in air before being stimulated with either IBMX (1 mm) or forskolin (5 μm) + IBMX (1 mm) for a further 10 min. Intracellular cAMP levels were determined by measuring the amount of [^3^H]‐cAMP in each sample. ***Significant effect of forskolin (*P* < 0.001; *P < 0.05); ^†^significant effect of hypercapnia (*P* < 0.05). Data represent mean ± SEM; *n* = 6 for each.

We first assessed the effect of hypercapnia on the pH_i_ of Calu‐3 cells as it is well known that raising CO_2_ generally induces cytosolic acidification. Cells were first perfused with Krebs solution gassed with 5% (v/v) CO_2_ to maintain cells in a normocapnic environment. Perfusing cells with 10% (v/v) CO_2_ caused pH_i_ to decrease by 0.18 ± 0.01 pH units (*n* = 60). This intracellular acidosis recovered after ∼20 min even upon continuous exposure of cells to 10% (v/v) CO_2_ (Fig. 1 *A*). We therefore chose 20 min as an appropriate time to study the effects of acute hypercapnia as cells would have recovered their pH_i_. Exposure of cells to 10% (v/v) CO_2_ for 20 min did not alter the integrity of the epithelial monolayer as assessed by recording TEER. In normocapnia, TEER was 671 ± 42Ω cm^−2^ (*n* = 3) and 600 ± 42Ω cm^−2^ in monolayers of Calu‐3 cells exposed to acute hypercapnia (*P* > 0.05 *vs*. normocapnia; *n* = 3). For all experiments, [HCO_3_^−^] in the Krebs solution was maintained at 25 mm in both normocapnia and hypercapnia. This was necessary to ensure that any effects of hypercapnia on cAMP signalling were due to CO_2_‐dependent effects on tmAC as opposed to effects of HCO_3_^−^ on sAC – an enzyme shown to be sensitive to HCO_3_^−^ (Chen *et al*. 2000). In addition, given the scope of our work was to investigate the effect of raised CO_2_ on bicarbonate secretion, changing [HCO_3_^−^] in hypercapnia would be predicted to compromise these studies.

As we have previously shown that cAMP signalling is sensitive to changes in CO_2_ (Townsend *et al*. 2009; Cook *et al*. 2012), [cAMP]_i_ was measured in conditions of normocapnia and after 20 min exposure to hypercapnia, with the incubation media buffered to pH 7.4 in each condition to control for differences in extracellular pH (pH_e_). In the presence of the non‐specific phosphodiesterase (PDE) inhibitor IBMX, there was no effect of hypercapnia on [cAMP]_i_ (Fig. 1 *B*). Stimulation of cells with the cAMP elevating agonist forskolin (added *after* 20 min of exposure to 5 or 10% CO_2_ to allow for pH_i_ recovery) produced a 3.3 ± 0.5‐fold increase in [cAMP]_i_ in normocapnia (*P* < 0.001; *n* = 6; Fig. 1 *B*) but this was significantly reduced to a 2.3 ± 0.4‐fold increase in [cAMP]_i_ in cells exposed to acute hypercapnia (*P* < 0.05 *vs*. normocapnia; *n* = 6; Fig. 1 *B*). When the cAMP levels produced in IBMX‐stimulated cells were subtracted from the cAMP levels measured in the presence of forskolin + IBMX, acute hypercapnia induced a 48 ± 4% reduction in [cAMP]_i_. These results demonstrate that cAMP signalling in Calu‐3 cells is responsive to elevated CO_2_, through a mechanism that is independent of changes in pH_e_ and not due to the CO_2_‐induced intracellular acidosis.

### Forskolin‐stimulated transepithelial anion secretion is reduced in conditions of acute hypercapnia in Calu‐3 cells

**Figure 2:**
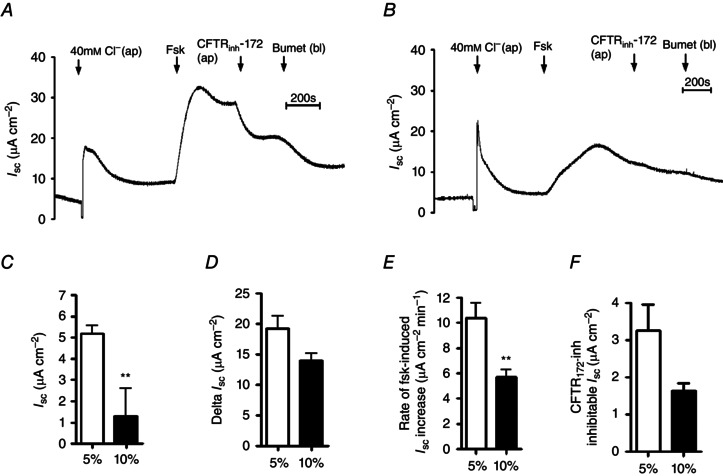
**Forskolin‐stimulated transepithelial anion secretion is reduced in conditions of acute hypercapnia in Calu‐3 cells** Calu‐3 cells were grown on permeable Snapwell supports and *I*_sc_ was measured using an Ussing chamber. *A*, a representative *I*_sc_ recording of a control experiment in which cells were exposed to 5% (v/v) CO_2_/95% (v/v) O_2_; *B*, a representative recording in which cells were pre‐exposed to 10% (v/v) CO_2_/90% (v/v) O_2_ for 20 min prior to being studied. Apical [Cl^−^] was reduced to 40 mm and cells were stimulated with forskolin (Fsk; 5 μm) before addition of apical CFTR_inh_‐172 (20 μm) and basolateral bumetanide (Bumet; 25 μm) as indicated. *C–F*, basal *I*_sc_ (*C*), maximal forskolin‐stimulated increase in *I*_sc_ (*D*), rate of increase in forskolin‐stimulated *I*_sc_ (*E*) and amount of forskolin‐stimulated current that was inhibitied by CFTR_inh_‐172 (*F*). **Significant effect of hypercapnia (*P* < 0.01). Data represent mean ± SEM; *n* = 10 for normocapnia and *n* = 8 for hypercapnia.

To assess whether the CO_2_‐induced reductions in forskolin‐stimulated [cAMP]_i_ modulated cAMP‐regulated transepithelial ion transport, *I*_sc_ measurements were made in monolayers of Calu‐3 cells. *I*_sc_ is the current required to clamp the transepithelial voltage difference (*V*_te_) to 0 mV. In Calu‐3 monolayers, the magnitude of *V*_te_ is mainly accounted for by transepithelial anion secretion (Lee *et al*. 1998; Devor *et al*. 1999; Cobb *et al*. 2003; Cuthbert *et al*. 2003; Shan *et al*. 2012) and therefore changes in *I*_sc_ reflect changes in anion secretion. Figure 2 *A* shows a representative recording of *I*_sc_ in normocapnic conditions. To maximise electrogenic Cl^−^ secretion, a basolateral to apical Cl^−^ gradient was applied across the monolayer by reducing apical [Cl^−^] to 40 mm by substitution of 84 mm NaCl with equimolar Na‐gluconate. In normocapnia, prior to reducing the apical Cl^−^ concentration, Calu‐3 cells displayed a basal *I*_sc_ of 5.2 ± 0.4 μA and further investigations showed that this basal *I*_sc_ was insensitive to both the basolateral Na^+^/K^+^/2Cl^−^ (NKCC1) inhibitor bumetanide (25 μm) and the NHE inhibitor Ethyl‐isopropyl amiloride (EIPA) (3 μm) (Masereel *et al*. 2003), whereas application of the CFTR blocker CFTR_inh_‐172 (20 μm) reduced basal *I*_sc_ by 48.5 ± 4.2% (*P* < 0.01; *n* = 3), indicating that the majority of basal *I*_sc_ was mediated by CFTR. Interestingly, in cells exposed to 20 min hypercapnia (Fig. 2 *B*), basal *I*_sc_ was reduced to 1.3 ± 1.3 μA (*P* < 0.01 *vs*. normocapnia; *n* = 8; Fig. 2 *C*), implying that acute hypercapnia inhibited CFTR‐dependent anion secretion under resting conditions. After establishing a basolateral to apical Cl^−^ gradient, addition of forskolin stimulated an increase in *I*_sc_ which peaked after approximately 90 s to a maximal level and then decreased slightly until a new steady state was reached. The forskolin‐stimulated increase in *I*_sc_ was blocked by a combination of apical CFTR_inh_‐172 (20 μm) and basolateral bumetanide (25 μm), and both the magnitude and the rate of *I*_sc_ increase were significantly reduced by 61.8 ± 16.0 and 73.4 ± 6.8%, respectively, by the PKA inhibitor H‐89 (*P* < 0.05 *vs*. control; *n* = 3). These results demonstrated that CFTR‐dependent anion secretion mediated the forskolin‐stimulated increase in *I*_sc_, consistent with previous studies (Welsh & Smith, 2001; Kreda *et al*. 2007; Shan *et al*. 2012). The maximal forskolin‐stimulated increase in *I*_sc_ (Δ*I*_sc_) was 19.3 ± 2.0 μA cm^−2^ (*n* = 10) in normocapnia compared to 14.1 ± 1.1 μA cm^−2^ in acute hypercapnia (*P* = 0.053 *vs*. normocapnia; *n* = 8; Fig. 2 *D*). The rate of forskolin‐stimulated increase in *I*_sc_ in normocapnia was 10.4 ± 1.3 μA cm^−2^ min^−1^ (*n* = 10), which was reduced to 5.7 ± 0.6 μA cm^−2^ min^−1^ (*P* < 0.01 *vs*. normocapnia; *n* = 8; Fig. 2 *E*) in cells exposed to acute hypercapnia. These results, combined with those in Fig. 1, imply that attenuation of forskolin‐stimulated cAMP levels by acute hypercapnia was sufficient to significantly reduce the rate of cAMP‐regulated anion secretion in Calu‐3 cells. In addition, the forskolin‐stimulated *I*_sc_ that was sensitive to CFTR_inh_‐172 was also measured. In normocapnia, this was 3.3 ± 0.7 μA cm^−2^ (*n* = 10) and although it was lower in hypercapnia (1.6 ± 0.2 μA cm^−2^; *n* = 8), this was not statistically significant, although a clear trend existed (*P* = 0.058 *vs*. normocapnia; Fig. 2 *F*). Taken together with the data displayed in Fig. 2 *C* and *E*, these findings suggest CFTR activity is lower in hypercapnia in both basal and forskolin‐stimulated conditions.

### Acute hypercapnia reduces adenosine but not IBMX‐stimulated transepithelial anion secretion in Calu‐3 cells

**Figure 3:**
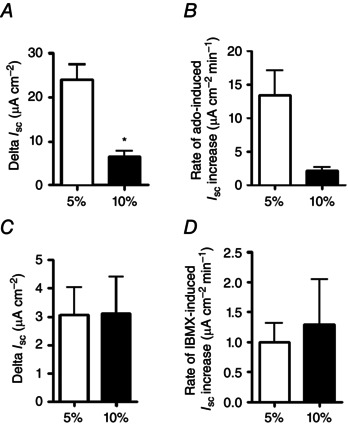
**Acute hypercapnia reduces adenosine but not IBMX‐stimulated transepithelial anion secretion in Calu‐3 cells** Calu‐3 cells were grown on permeable Snapwell supports and *I*_sc_ was measured using an Ussing chamber. For control experiments, cells were gassed with 5% (v/v) CO_2_/95% (v/v) O_2_ whilst hypercapnia was induced by pre‐exposing cells to 10% (v/v) CO_2_/90% (v/v) O_2_ for 20 min prior to being studied. Apical [Cl^−^] was reduced to 40 mm and cells were stimulated with either adenosine (10 μm) or IBMX (1 mm) before addition of apical CFTR_inh_‐172 (20 μm) and basolateral bumetanide (25 μm). *A*, the maximal adenosine‐stimulated increase in *I*_sc_; *B*, the rate of increase in adenosine‐stimulated *I*_sc_. *Significant effect of hypercapnia (*P* < 0.05). Data represent mean ± SEM; *n* = 5 for normocapnia and *n* = 3 for hypercapnia. *C*, the maximal IBMX‐stimulated increase in *I*_sc_; *D*, the rate of increase in IBMX‐stimulated *I*_sc_. Data represent mean ± SEM; *n* = 3 for normocapnia and *n* = 4 for hypercapnia.

Having shown that hypercapnia reduced forskolin‐stimulated *I*_sc_ in Calu‐3 cells, it was important to investigate whether hypercapnia also elicited similar effects when a more physiological agonist was used to increase [cAMP]_i_ in Calu‐3 cells. For this, cells were stimulated with adenosine (Cobb *et al*. 2003) and the resulting *I*_sc_ was measured. In normocapnia, adenosine stimulated a maximal *I*_sc_ increase of 23.9 ± 3.5 μA cm^−2^ (*n* = 5), which was significantly reduced to 6.4 ± 1.4 μA cm^−2^ in cells exposed to acute hypercapnia (*P* < 0.05 *vs*. normocapnia; *n* = 3; Fig. 3 *A*). The rate of the adenosine‐stimulated increase in *I*_sc_ was 13.4 ± 8.4 μA cm^−2^ min^−1^ (*n* = 5) in normocapnia which was reduced to 2.3 ± 0.8 μA cm^−2^ min^−1^ in acute hypercapnia (*P* = 0.06 *vs*. normocapnia; *n* = 3; Fig 3 *B*). Therefore, these data demonstrated that hypercapnia reduced adenosine‐stimulated, CFTR‐dependent anion secretion in Calu‐3 cells, which mimicked what was observed with forskolin. Interestingly, when [cAMP]_i_ levels were increased by stimulation of cells with IBMX, there was no effect of acute hypercapnia on either the IBMX‐stimulated Δ*I*_sc_ (normocapnia = 3.1 ± 0.9 μA cm^−2^; hypercapnia = 3.1 ± 1.3 μA cm^−2^; *P* > 0.05 *vs*. normocapnia; *n* = 3–4; Fig. 3 *C*) or the rate of IBMX‐stimulated increase in *I*_sc_ (normocapnia = 1.0 ± 0.31 μA cm^−2^ min^−1^; hypercapnia = 1.2 ± 0.8 μA cm^−2^ min^−1^; *P* > 0.05 *vs*. normocapnia; *n* = 3–4; Fig. 3 *D*). Therefore, these data support the observations in Fig. 1 *B*, which demonstrated that IBMX‐stimulated increases in [cAMP]_i_ was insensitive to CO_2_, and suggest hypercapnia‐induced changes in [cAMP]_i_ were not due to modulation of IBMX‐sensitive PDE activity.

### The effect of hypercapnia on cAMP‐dependent transepithelial anion secretion is independent of CO_2_‐induced intracellular acidosis

**Figure 4:**
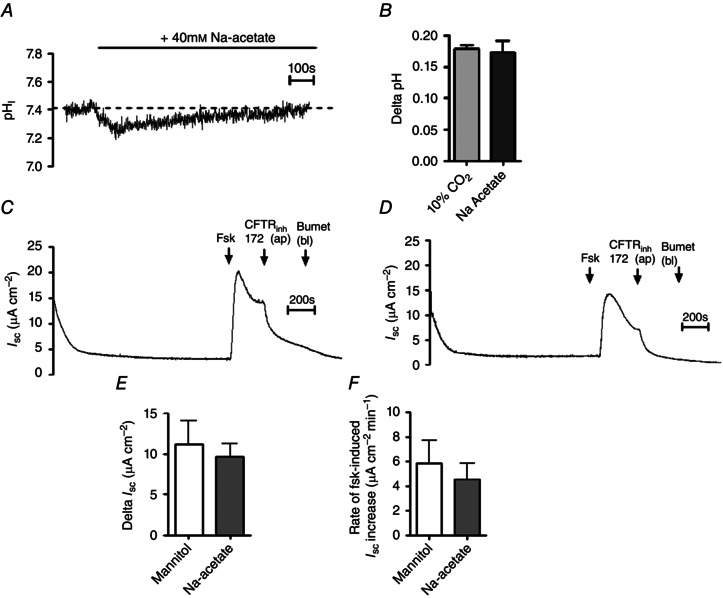
**The effect of hypercapnia on cAMP‐dependent transepithelial anion secretion is independent of CO_2_‐induced intracellular acidosis** *A*, a representative experiment in which Calu‐3 cells were gassed with 5% (v/v) CO_2_/95% (v/v) O_2_ and exposed to 40 mm sodium acetate and pH_i_ was measured using fluorescence microscopy. *B*, summary of the magnitude of the intracellular acidosis resulting from either 10% CO_2_ or sodium acetate. Data represent mean ± SEM; *n* = 60 for 10% CO_2_ and *n* = 6 for sodium acetate. *C* and *D*, representative *I*_sc_ measurements in which cells were exposed to 80 mm mannitol or 40 mm sodium acetate, respectively, for 20 min prior to addition of forskolin (Fsk; 5 μm), apical CFTR_inh_‐172 (20 μm) and basolateral bumetanide (Bumet; 25 μm) as indicated. *E* and *F*, summary of the effect of sodium acetate on the magnitude and the rate, respectively, of the forskolin‐stimulated increase in *I*_sc_. Data represent mean ± SEM, *n* = 5 for each.

Although *I*_sc_ measurements performed in hypercapnia were made after 20 min of exposure to 10% CO_2_, during which time pH_i_ had recovered from intracellular acidosis (see Fig. 1 *A*), it was possible the intracellular acidosis may have induced long‐term modifications to transporters involved in cAMP‐regulated anion secretion. Therefore, cells were acid loaded using 40 mm sodium acetate, which caused an intracellular acidification of 0.17 ± 0.02 (*n* = 6) that recovered within 20 min (Fig. 4 *A* and *B*) and was thus highly similar to the effect of 10% CO_2_. Thus, the effect of forskolin on *I*_sc_ was measured in cells exposed to 40 mm sodium acetate or 80 mm mannitol (to compensate for the increased osmolarity of the sodium acetate‐containing solutions). Representative experiments are shown in Fig. 4 *C* and *D*. There was no effect of 40 mm sodium acetate on either the magnitude or the rate of forskolin‐stimulated increases in *I*_sc_ (Fig. 4 *E* and *F*), therefore demonstrating that the CO_2_‐induced intracellular acidosis does not contribute to the effects of hypercapnia on cAMP‐stimulated anion transport in Calu‐3 cells.

### Surface expression of CFTR is unaffected by hypercapnia

**Figure 5:**
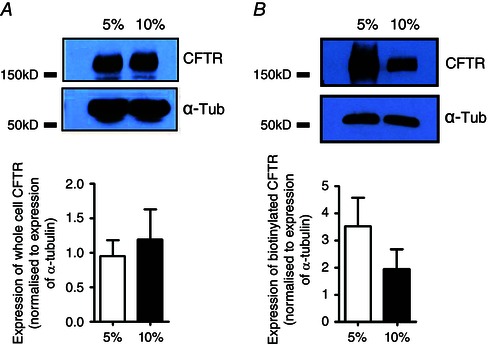
**Cell surface expression of CFTR is unaffected by acute hypercapnia** Calu‐3 cells were grown on permeable transwell supports and membrane expression of CFTR was assessed using a biotinylation assay. *A*, an example blot of whole cell CFTR expression under 5% CO_2_ and 10% CO_2_ and the relative expression of whole cell CFTR when normalised to expression of whole cell α‐tubulin. Data represent mean ± SEM; *n* = 5. *B*, an example blot of biotinylated CFTR expression, used as a marker of surface expression, under 5% CO_2_ and 10% CO_2_ and the relative expression of biotinlayed CFTR when normalised to expression of biotinylated α‐tubulin. Data represent mean ± SEM; *n* = 4.

Our results from the *I*_sc_ measurements indicated that CO_2_‐induced reductions in [cAMP]_i_ were sufficient to reduce cAMP‐stimulated, CFTR‐dependent anion secretion in Calu‐3 cells. To investigate if this observation was due to the effect of CO_2_ on cAMP and not on cell surface levels of CFTR, the amount of CFTR present at the apical membrane was assessed by cell surface biotinylation. Figure 5 shows that after normalising CFTR levels to α‐tubulin, there was no significant effect of CO_2_ on either total cell CFTR expression (*P* > 0.05; *n* = 5, Fig. 5 *A*) or cell surface CFTR expression (*P* > 0.05; *n* = 4, Fig. 5 *B*), which therefore suggest that mechanisms which control CFTR expression at the plasma membrane are insensitive to hypercapnia.

### CFTR‐regulated, pendrin‐dependent apical HCO_3_^−^ secretion is unaffected by hypercapnia

**Figure 6:**
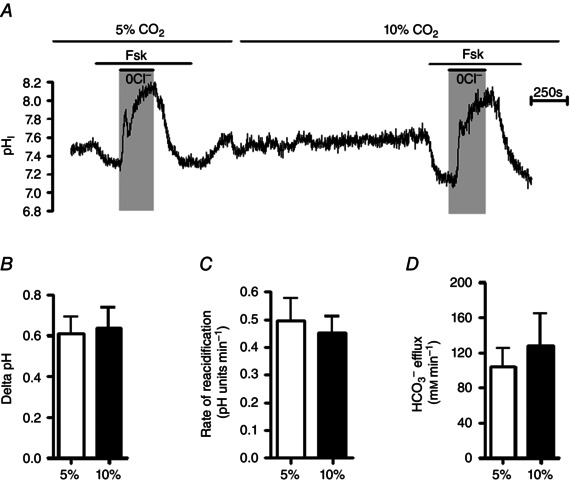
**CFTR‐regulated, pendrin‐dependent apical HCO_3_^−^ efflux is unaffected by hypercapnia** *A*, a representative pH_i_ experiment in which the effect of acute hypercapnia on 5 μm forskolin‐stimulated, CFTR‐regulated apical HCO_3_^−^ transport was assessed by removal and subsequent re‐addition of apical Cl^−^. *B*, ΔpH in response to removal of Cl^−^. *C* and *D*, rate of re‐acidification (*C*) and HCO_3_^−^ flux resulting from re‐addition of apical Cl^−^ (*D*). Data represent mean ± SEM; *n* = 6 for each.

Having identified that hypercapnia reduces cAMP‐stimulated anion secretion in Calu‐3 cells, it was interesting to assess whether CO_2_ was modulating Cl^−^ or HCO_3_^−^ secretion or indeed both. pH_i_ measurements were performed to indirectly measure HCO_3_^−^ transport across the cells. At the apical membrane, we have previously shown that Calu‐3 cells express the Cl^−^/HCO_3_^−^ anion exchanger pendrin, which mediates the majority of HCO_3_^−^ efflux from the cell (Garnett *et al*. 2011). Pendrin activity was also shown to be regulated by CFTR. To measure CFTR‐dependent pendrin activity, cells were stimulated with forskolin and pendrin activity was assessed by Cl^−^ removal and re‐addition (Fig. 6 *A*) (Garnett *et al*. 2011). In normocapnia, removal of apical Cl^−^ caused pH_i_ to increase by 0.61 ± 0.08 units (*n* = 6), due to reversal of pendrin‐mediated Cl^−^/HCO_3_^−^ exchange, whilst in hypercapnia this increase in pH_i_ was 0.64 ± 0.10 (*P* > 0.05 *vs*. normocapnia; *n* = 6, Fig. 6 *B*). Furthermore, reintroduction of apical Cl^−^ caused pH_i_ to re‐acidify at a rate of 0.49 ± 0.08 pH units min^−1^ in normocapnia and 0.45 ± 0.06 pH units min^−1^ in hypercapnia (*P* > 0.05; *n* = 6; Fig. 6 *C*) which equated to an HCO_3_^−^ efflux of 104 ± 21 mm HCO_3_^−^ min^−1^ and 127 ± 38 mm HCO_3_^−^ min^−1^, respectively (*P* > 0.05; *n* = 6; Fig. 6 *D*). Note that in forskolin‐stimulated conditions, the basolateral anion exchanger, AE2, was almost completely inhibited, both in normocapnia (96.9 ± 1.9% inhibition; *n* = 4) and in hypercapnia (93.8 ± 4.3% inhibition; *n* = 4), consistent with previous findings (Garnett *et al*. 2011). Thus, AE2‐dependent HCO_3_^−^ transport can be eliminated from having any effect on these measurements. Therefore, these data show that apical CFTR‐dependent anion exchange activity was unaffected by acute hypercapnia and suggested that HCO_3_^−^ transport across the apical membrane was insensitive to changes in CO_2_.

### Acute hypercapnia does not alter cAMP‐stimulated NBC activity in Calu‐3 cells

**Figure 7:**
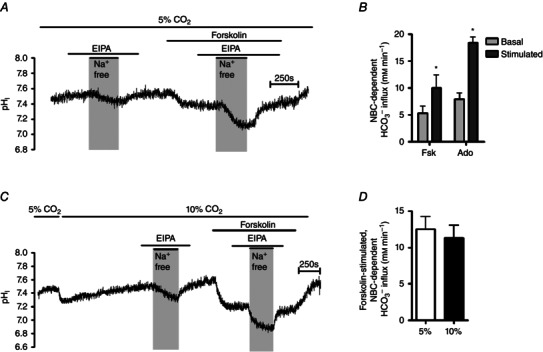
**Hypercapnia does not alter cAMP‐stimulated NBC activity in Calu‐3 cells** *A*, a representative pH_i_ experiment in which NBC activity was assessed under basal and forskolin‐stimulated conditions in 5% CO_2_. EIPA (3 μm) was present to inhibit the NHE. *B*, the effect of the cAMP agonists forskolin (5 μm) and adenosine (10 μm) on NBC‐dependent HCO_3_^−^ influx. *Significant effect of agonist stimulation (*P* < 0.05). Data represent mean ± SEM; *n* = 3 for each. *C*, a representative pH_i_ experiments in which forskolin‐stimulated NBC activity was assessed in conditions of acute hypercapnia. EIPA (3 μm) was present to inhibit the NHE. *E*, the effect of hypercapnia on forskolin‐stimulated NBC activity. Data represent mean ± SEM; *n* = 7 for each.

To investigate HCO_3_^−^ transport across the basolateral membrane, we measured the activity of NBC transporters, which have been shown to mediate basolateral membrane HCO_3_^−^ import in Calu‐3 cells (Lee *et al*. 1998; Devor *et al*. 1999; Shan *et al*. 2012). NBC activity was monitored by measuring changes in pH_i_ following the removal of basolateral Na^+^ (to inhibit NBC) and the re‐addition of basolateral Na^+^ (to re‐activate NBC), as described by Yang *et al*. (2009), in the presence of EIPA to inhibit NHE activity. However, it was first necessary to determine whether NBC activity in Calu‐3 cells was cAMP‐dependent. Figure 7 *A* and *B* shows that both forskolin and adenosine stimulated a 2.3 ± 0.4‐fold (*n* = 3; *P* < 0.05) and 2.5 ± 0.5‐fold (*n* = 3; *P* < 0.05) increase, respectively, in NBC activity, under normocapnic conditions, indicating that NBC activity in Calu‐3 cells is increased by cAMP. The effect of acute hypercapnia on cAMP‐regulated NBC activity was next assessed. Here, NBC activity was measured in normocapnic conditions (Fig. 7 *A*) or after cells had been exposed to 20 min of hypercapnia (Fig. 7 *C*). As summarised in Fig. 7 *D*, forskolin stimulated an NBC‐dependent HCO_3_^−^ influx of 12.5 ± 1.8 mm min^−1^ (*n* = 7) under normocapnia whilst in hypercapnia, forskolin‐stimulated NBC‐dependent HCO_3_^−^ influx was 11.3 ± 1.7 mm min^−1^ (*n* = 7; *P* > 0.05 *vs*. normocapnia). These findings suggest that, like pendrin, acute hypercapnia does not affect cAMP‐stimulated NBC activity and thus imply that CO_2_‐induced effects on cAMP‐regulated anion transport were not due to changes in HCO_3_^−^ secretion *per se* and suggested only Cl^−^ secretion was sensitive to elevated CO_2_.

### Hypercapnia reduces the volume of forskolin‐stimulated fluid secretion in Calu‐3 cells but has no effect on pH

**Figure 8:**
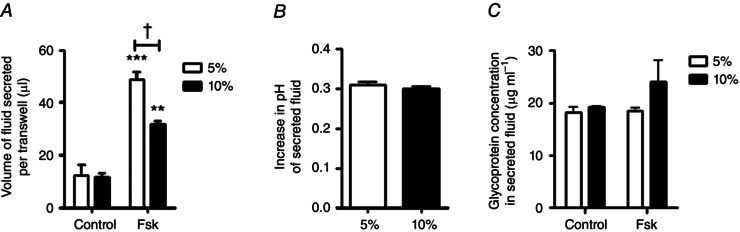
**Hypercapnia reduces the volume of forskolin‐stimulated fluid secretion in Calu‐3 cells** Cells were stimulated with forskolin (Fsk; 5 μm) and incubated for 24 h in either 5% CO_2_ (v/v) in air or 10% CO_2_ (v/v) in air in high Cl^−^ Krebs solution at 37°C. *A*, the effect of chronic hypercapnia on the volume of fluid secreted over 24 h. **Significant effect of forskolin stimulation compared to unstimulated control cells (*P* < 0.01; ***P < 0.001); †significant effect of 10% CO_2_ (*P* < 0.05). Data represent mean ± SEM; *n* = 3 for each. *B*, the increase in pH of forskolin‐stimulated secreted fluid relative to unstimulated control cells. Data represent mean ± SEM; *n* = 3 for each. *C*, the effects of forskolin and hypercapnia on the amount of glycoprotein present in the secreted fluid, quantified by the PAS assay. Data represent mean ± SEM; *n* = 3 for each.

We have previously shown that stimulation of Calu‐3 cells with forskolin for 24 h increased the secretion of a HCO_3_^−^‐rich fluid. Furthermore, based on pharmacological and genetic knockdown experiments, we suggested that cAMP‐stimulated liquid secretion was primarily regulated by CFTR, while HCO_3_^−^ secretion was not directly *via* CFTR but through Cl^−^/HCO_3_^−^ *via* pendrin (Garnett *et al*. 2011, 2013). Given that it appears separate transporters were responsible for Cl^−^ and HCO_3_^−^ secretion in Calu‐3 cells, it was of interest to assess if hypercapnia impacted upon forskolin‐stimulated ion and fluid secretion. Calu‐3 cells were stimulated with forskolin in either 5% CO_2_ (v/v) in air or 10% CO_2_ (v/v) in air for 24 h and the amount and pH of the secreted fluid were analysed. Note that TEER was not significantly different between normocapnic controls (682 ± 28 Ω cm^−2^; *n* = 6) and cells incubated for 24 h in hypercapnia (681 ± 6 Ω cm^−2^; *P* > 0.05 *vs*. control; *n* = 6), suggesting that chronic hypercapnia did not alter tight junction properties of Calu‐3 cells. In normocapnic conditions, unstimulated cells secreted 12 ± 4 μl fluid over 24 h (*n* = 3), which was significantly enhanced 3.9 ± 0.2‐fold to 49 ± 3 μl by forskolin stimulation (*P* < 0.01 *vs*. unstimulated cells; *n* = 3; Fig. 8 *A*). In hypercapnic conditions, unstimulated cells secreted 12 ± 1 μl fluid over 24 h which was almost identical to that seen in normocapnia (*P* > 0.05; *n* = 3). However, although forskolin increased fluid secretion to 32 ± 1 μl over 24 h (*P* < 0.01; *n* = 3; Fig. 8 *A*), this 2.7 ± 0.1‐fold increase in the volume of forskolin‐stimulated fluid secretion was significantly lower than that observed in normocapnia (*P* < 0.05 *vs*. normocapnia; *n* = 3; Fig. 8 *A*). This suggested chronic hypercapnia impaired cAMP‐regulated CFTR‐dependent Cl^−^ secretion in airway epithelia to reduce the osmotic driving force for fluid secretion. The pH of the secreted fluid was also measured. In normocapnia, the pH of secreted fluid increased from 7.52 ± 0.01 to 7.82 ± 0.06 (*P* < 0.01; *n* = 3) indicative of a greater [HCO_3_^−^] in forskolin‐stimulated fluid secretion. This pH increase of 0.31 ± 0.01 was not different from the pH increase of 0.30 ± 0.01 observed in hypercapnia (7.21 ± 0.04 to 7.51 ± 0.02; *P* < 0.01 *vs*. unstimulated controls; *P* > 0.05 *vs*. normocapnia; *n* = 3; Fig. 8 *B*) with the lower pH values observed due to acidosis induced by elevated CO_2_. Using the Henderson–Hasselbalch equation to calculate [HCO_3_^−^] revealed that the forskolin‐stimulated fluid contained 61.6 ± 9.5 mm HCO_3_^−^ in normocapnia, which was not significantly different from the 58.2 ± 2.4 mm HCO_3_^−^ in the forskolin‐stimulated fluid in hypercapnia (*P* > 0.05; *n* = 3). Together, these findings suggest that CFTR‐dependent electrogenic Cl^−^ secretion is CO_2_‐sensitive, whilst pendrin‐dependent HCO_3_^−^ secretion is CO_2_‐insenstive, and supports the findings from *I*_sc_ and pH_i_ measurements (Figs 2, 6 and 7). In addition as mucin secretion has been shown to be dependent on [HCO_3_^−^] (Garcia *et al*. 2009; Chen *et al*. 2010; Gustafsson *et al*. 2012; Ridley *et al*. 2014), we also analysed the glycoprotein content of the secreted fluid by the PAS assay. In normocapnia, forskolin did not alter the amount of glycoproteins detected relative to unstimulated cells (18.5 ± 0.5 *vs*. 18.2 ± 1.0 μg ml^−1^, respectively; *P* > 0.05; *n* = 3; Fig. 8 *C*). Furthermore, hypercapnia had no effect on glycoprotein secretion from Calu‐3 cells relative to normocapnia in either basal or forskolin‐stimulated cells. Unstimulated cells secreted 19.2 ± 0.1 μg ml^−1^ glycoprotein (*P* > 0.05 *vs*. unstimulated cells in normocapnia; *n* = 3), which was unchanged in response to forskolin stimulation (24.0 ± 4.0 μg ml^−1^; *P* > 0.05 *vs*. unstimulated cells in hypercapnia; *P* > 0.05 *vs*. stimulated cells in normocapnia; *n* = 3; Fig. 8 *C*). Therefore, hypercapnia modulated transporters involved in regulating the volume of secreted fluid but not those involved in mediating its composition.

### Hypercapnia reduces forskolin‐stimulated increases in *I*_sc_ across primary human bronchial epithelial cells

**Figure 9:**
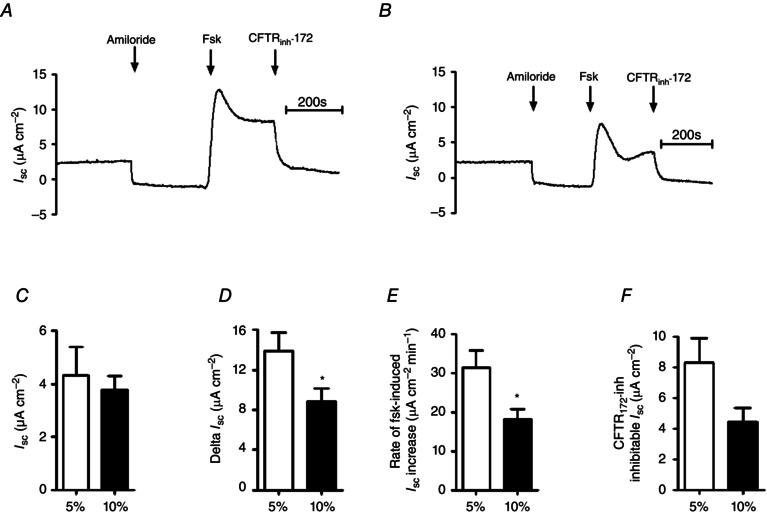
**Forskolin‐stimulated transepithelial anion secretion is reduced in conditions of acute hypercapnia in primary human bronchial epithelial cells** Primary human bronchial epithelial cells were grown on collagen‐coated permeable Snapwell supports and allowed to differentiate at an ALI for 30–35 days before *I*_sc_ was measured using an Ussing chamber. *A*, a representative *I*_sc_ recording of a control experiment in which cells were exposed to 5% (v/v) CO_2_/95% (v/v) O_2_; *B*, a representative recording in which cells were pre‐exposed to 10% (v/v) CO_2_/90% (v/v) O_2_ for 20 min prior to being studied. Apical [Cl^−^] and basolateral [Cl^−^] were both 124 mm for these experiments. Cells were treated with apical amiloride (Amil; 10 μm) and stimulated with forskolin (Fsk; 10 μm) before addition of apical CFTR_inh_‐172 (20 μm) as indicated. *C–F*, basal *I*_sc_ (*C*), maximal forskolin‐stimulated increase in *I*_sc_ (*D*), rate of increase in forskolin‐stimulated *I*_sc_ (*E*) and amount of forskolin‐stimulated current that was inhibitied by CFTR_inh_‐172 (*F*). *Significant effect of hypercapnia (*P* < 0.05). Data represent mean ± SEM; *n* = 6 for each.

To assess whether hypercapnia elicited similar effects in primary airway epithelia as it did in an airway epithelial cell line, *I*_sc_ measurements were made on fully differentiated primary human bronchial epithelial cells (HBECs) grown under ALI. Figure 9 *A* and *B* shows representative experiments performed in conditions of normocapnia and hypercapnia, respectively. Hypercapnia had no effect on basal *I*_sc_ (4.3 ± 1.1 μA cm^−2^ in normocapnia and 3.8 ± 0.5 μA cm^−2^ in acute hypercapnia; *P* > 0.05 *vs*. normocapnia; *n* = 6; Fig. 9 *C*). However, it was found that the basal *I*_sc_ was sensitive to apical amiloride (10 μm), which reduced basal *I*_sc_ by 5.0 ± 0.9 μA cm^−2^ in normocapnia (*n* = 6) and 4.4 ± 0.6 μA cm^−2^ in hypercapnia (*P* > 0.05 *vs*. normocapnia; *n* = 6), indicating that these cells expressed functional epithelial Na^+^ channels (ENaC). Stimulation of cells with forskolin in normocapnia induced a maximal increase in *I*_sc_ of 13.9 ± 1.8 μA cm^−2^ (*n* = 6) which was significantly reduced to 8.8 ± 1.3 μA cm^−2^ in cells that had been exposed to acute hypercapnia (*P* < 0.05 *vs*. normocapnia; *n* = 6; Fig. 9 *D*). Furthermore, the rate of forskolin‐stimulated *I*_sc_ increase was also significantly reduced from 31.3 ± 4.4 μA cm^−2^ min^−1^ (*n* = 6) in normocapnia to 18.1 ± 2.6 μA cm^−2^ min^−1^ in hypercapnia (*P* < 0.05 *vs*. normocapnia; *n* = 6; Fig. 9 *E*). These data are consistent with the findings from Calu‐3 cells and suggest that hypercapnia reduces cAMP‐stimulated CFTR‐dependent anion transport in primary human airway epithelial cells as well as in an airway epithelia cell line. When measuring the amount of CFTR_inh_‐172‐sensitive current, it was again found that there was a clear trend for this to be lower in acute hypercapnia, supporting the findings that CFTR activity was reduced by 10% CO_2_. As shown in Fig. 9 *F*, in normocapnia, forskolin‐stimulated CFTR_inh_‐172‐sensitive current was 8.3 ± 1.6 μA cm^−2^ and was reduced in hypercapnia to 4.4 ± 0.9 μA cm^−2^ (*n* = 6; *P* > 0.05 *vs*. normocapnia; Fig. 9 *F*).

## Discussion

The ability of CO_2_ to act as a cell signalling molecule is currently gaining substantial support within human physiology. Here we show, for the first time, that hypercapnia modulates cAMP‐dependent signalling, as well as cAMP‐dependent ion and fluid transport, in both a human airway epithelial cell line and also in primary HBECs. We found that acute hypercapnia caused a significant reduction in forskolin‐stimulated [cAMP]_i_ levels in Calu‐3 cells – even in the presence of a PDE inhibitor – which was independent of CO_2_‐induced intracellular or extracellular acidosis (Fig. 1 *B*). Interestingly, hypercapnia did not affect cAMP levels in cells stimulated with IBMX only (Fig. 1 *B*), implying that the CO_2_‐induced attenuation of [cAMP]_i_ was not due to modulation of PDE activity, consistent with our previous results (Townsend *et al*. 2009; Cook *et al*. 2012). The apparent lack of effect of hypercapnia in the absence of forskolin suggests that for hypercapnia to alter tmAC activity, the cyclase needs to be in an active state. Zhang *et al*. (1997) have described the presence of hydrophobic forskolin binding pockets on tmAC, and forskolin binding at these sites induces a conformational change leading to dimerisation of the two catalytic subunits of tmAC. Thus, it seems likely that CO_2_ can only modulate tmAC activity when it is held within this ‘forskolin‐bound’ state. Similar conformational changes in tmAC are induced when free G_αs_ bind to the enzyme, implying that CO_2_ modulates tmAC activity *via* the same mechanism when cells are stimulated with G‐protein coupled receptor agonists such as adenosine (Tesmer *et al*. 1997).

The hypercapnic‐induced reduction in forskolin‐stimulated cAMP levels also had significant effects on forskolin‐stimulated transepithelial ion transport in Calu‐3 cells. In the presence of a basolateral to apical Cl^−^ gradient, 10% CO_2_ caused an ∼45% reduction in the rate of forskolin‐stimulated increase in CFTR_inh_‐172 and bumetanide‐sensitive *I*_sc_ (Fig. 2 *E*). These findings imply that CO_2_‐induced changes in [cAMP]_i_ were sufficient to reduce CFTR‐dependent electrogenic anion secretion in Calu‐3 cells. Hypercapnia also produced the same effect when cells were stimulated with the physiological cAMP agonist adenosine but did not alter IBMX‐stimulated changes in *I*_sc_ (Fig. 3). These findings indicated that CO_2_‐dependent reductions in [cAMP]_i_ were a result of modulations to tmAC‐dependent cAMP production as opposed to PDE‐dependent cAMP breakdown, which supports previous findings from our laboratory (Townsend *et al*. 2009; Cook *et al*. 2012). We were also able to conclude that the modulations to cAMP‐regulated anion transport in hypercapnia were not a result of the CO_2_‐induced intracellular acidosis as mimicking this acid load using sodium acetate did not alter forskolin‐stimulated increases in *I*_sc_ (Fig. 4).

Biotinylation experiments further showed that the effect of hypercapnia on *I*_sc_ could not be explained by a reduction in surface levels of CFTR (Fig. 5). These findings support our hypothesis that in cAMP‐stimulated conditions, the effects of CO_2_ were due to modulation of [cAMP]_i_ as opposed to CO_2_‐dependent effects on pathways involved in regulating CFTR surface expression, for instance endocytosis. Furthermore, these findings are of particular relevance given that hypercapnia has been shown to modulate the surface expression of the Na^+^/K^+^‐ATPase in mammalian alveolar epithelia (Briva *et al*. 2007), which therefore suggests that CO_2_ only induces endocytosis of specific ion transporters. Acute hypercapnia also significantly lowered basal *I*_sc_ in Calu‐3 cells. That a large component of this basal *I*_sc_ was sensitive to CFTR_inh_‐172 suggests that hypercapnia also reduced the activity of CFTR under these conditions. However, hypercapnia did not alter levels of [cAMP]_i_ under resting conditions (Fig. 1 *B*), and hypercapnia did not alter surface CFTR expression (Fig. 5), indicating that the effect of high CO_2_ on resting CFTR activity was independent of its effects on cAMP and not due to loss of CFTR at the plasma membrane. Therefore, why we observed a decrease in basal *I*_sc_ in Calu‐3 cells exposed to acute hypercapnia remains unclear, but we cannot exclude the possibility that hypercapnia may have effects on basal [cAMP]_i_ that cannot be detected using our current method of quantification. Note that whilst hypercapnia induces a reversible intracellular acidosis (Fig. 1 *A*) and that CFTR has been shown to be pH‐sensitive (Reddy *et al*. 1998; Chen *et al*. 2009; Melani *et al*. 2010), the 10% CO_2_‐induced acidosis of ∼0.2 units is unlikely to significantly alter CFTR activity based on single channel recordings of CFTR expressed in mammalian cells (Chen *et al*. 2009) and measurements of CFTR‐dependent Cl^−^ conductance made in human sweat ducts (Reddy *et al*. 1998). Furthermore, the fact that all measurements of cAMP‐stimulated CFTR activity were made after cells had recovered pH_i_ in response to CO_2_‐induced acidosis also strongly argues against any pH_i_‐dependent effects on CFTR activity in hypercapnia.

To identify the transport of which anion (Cl^−^ or HCO_3_^−^) hypercapnia was modulating, pH_i_ measurements were performed to indirectly measure HCO_3_^−^ transport in real time in polarised cultures of Calu‐3 cells. Importantly, we showed that cAMP‐stimulated, pendrin‐dependent apical HCO_3_^−^ secretion and cAMP‐stimulated, NBC‐dependent basolateral HCO_3_^−^ influx were both insensitive to hypercapnia (Figs 6 and 7), suggesting that hypercapnia did not alter HCO_3_^−^ transport directly in Calu‐3 cells. Thus, the results from the *I*_sc_ measurements suggested that the CO_2_‐induced reduction in electrogenic anion secretion was specifically due to a reduction in transepithelial Cl^−^ secretion. Thus, it appears that cAMP‐regulated transporters have different sensitivities to CO_2_‐induced decreases in [cAMP]_i_ in Calu‐3 cells. Although the reasons for this are unclear at the present, it is known that CFTR exists in a microdomain at the apical membrane of airway epithelial cells, in which cAMP signalling is highly compartmentalised (Barnes *et al*. 2005; Penmatsa *et al*. 2010). A decrease in cAMP levels in such a compartmentalised microdomain would have more pronounced effects than in areas of the cell where cAMP signalling is less compartmentalised, for instance at the basolateral subcellular location. Similarly, apical and basolateral microdomains may possess distinct tmAC isoforms that could display differential sensitivities to raised CO_2_.

We also observed similar results when investigating the effects of hypercapnia on cAMP‐stimulated anion and fluid transport using a different approach. Incubating cells for 24 h in hypercapnia enabled us to assess the effect of hypercapnia on the volume, as well as the composition, of the secreted fluid (Fig. 8). We found that hypercapnia did not affect the amount of fluid secreted under basal conditions. This is consistent with results from Fig. 1 *B* that demonstrated cAMP levels in non‐stimulated Calu‐3 cells were insensitive to hypercapnia. However, the fluid secretion data do contradict our *I*_sc_ measurements in which CFTR_inh_‐172‐sensitive basal *I*_sc_ was reduced in hypercapnia, suggesting that CFTR may be altered by hypercapnia through a cAMP‐independent mechanism. Nonetheless, hypercapnia caused a significant reduction in the amount of secreted fluid under forskolin‐stimulated conditions (Fig. 8 *A*). We have previously shown that the volume of forskolin‐stimulated fluid secretion is predominantly mediated by electrogenic CFTR‐dependent Cl^−^ secretion (Garnett *et al*. 2011), strongly suggesting that hypercapnia reduced fluid secretion *via* an effect on CFTR‐dependent Cl^−^ transport. This was probably due to the CO_2_‐induced reduction in forskolin‐stimulated cAMP levels (Fig. 1 *B*). Although we demonstrated chronic hypercapnia did not affect the transepithelial resistance of Calu‐3 monolayers, indicating paracellular ion and fluid transport was not altered by 10% CO_2_, one cannot rule out the possibility that hypercapnia may alter the water permeability of the epithelial monolayer, which would be another interesting effect of elevated CO_2_. However, unpublished findings from our laboratory have found that the osmolarity of secreted fluid in Calu‐3 cells is unchanged in forskolin‐stimulated cells compared to control cells. Thus, as we know forskolin to increase ion and fluid secretion in Calu‐3 cells, these findings demonstrate that changes in transepithelial ion secretion do not alter water permeability and thus are unlikely to contribute to the changes in fluid secretion observed in hypercapnia. Kim *et al*. (2014) also suggest water permeability is unchanged in Calu‐3 cells even in conditions where ion secretion is stimulated. Interestingly, the [HCO_3_^−^] of forskolin‐stimulated fluid secretion was unaffected by chronic hypercapnia (Fig. 8 *B*). Garnett *et al*. (2011) demonstrated that the pH of forskolin‐secreted fluid was predominately regulated by the Cl^−^/HCO_3_^−^ exchanger pendrin, and not directly by CFTR, as fluid pH was insensitive to GlyH‐101 or genetic knockdown of CFTR, but was reduced by pendrin *K*_D_. Thus, our results demonstrate that CFTR and pendrin exhibit differential sensitivities to CO_2_. In addition, neither forskolin nor hypercapnia had any effect on the amount of glycoprotein detected in apical secretions from Calu‐3 cells, suggesting that neither treatment modified mucus secretion. Kreda *et al*. (2007) demonstrated that secretion of mucins by Calu‐3 cells, including MUC5AC, was a result of Ca^2+^‐dependent exocytosis of mucin granules, probably explaining why forskolin did not alter mucus secretion. Furthermore, these findings also imply that hypercapnia does not alter Ca^2+^‐dependent mucin secretion and therefore only modulates cAMP‐regulated responses.

Finally, the findings of acute hypercapnia on CFTR‐dependent *I*_sc_ in Calu‐3 cells were also replicated in fully differentiated HBECs. In these cells, 10% CO_2_ also significantly reduced cAMP‐stimulated CFTR‐dependent anion transport (Fig. 9). Although we did not measure [cAMP]_i_ in response to hypercapnia in HBECs, the ∼42% decrease in the rate of forskolin‐stimulated *I*_sc_ increase in HBECs was comparable to the ∼45% decrease observed in Calu‐3 cells, and thus suggests CO_2_ elicited its effects *via* similar mechanisms in both cell types. However, one interesting difference was the fact that hypercapnia had no effect on basal *I*_sc_ in HBECs whereas it did in Calu‐3 monolayers (see Figs 2 *C* and 9 *C*), suggesting that basal CFTR activity is less sensitive to CO_2_ in primary airway epithelia. However, basal *I*_sc_ in Calu‐3 cells was amiloride‐insensitive (our unpublished observations), as opposed to the large component of basal *I*_sc_ in HBECs that was inhibited by amiloride, suggesting different transporters regulate basal *I*_sc_ in the two cell types and probably explaining the differences in response to hypercapnia. Furthermore, given there was no effect of CO_2_ on amiloride‐sensitive *I*_sc_ in HBECs, ENaC activity was probably insensitive to acute hypercapnia. This reinforces the findings that acute hypercapnia mediates specific effects on CFTR as opposed to other membrane ion transporters.

In summary, we have shown for the first time that acute hypercapnia reduced cAMP production as well as cAMP‐stimulated, CFTR‐dependent Cl^−^, but not HCO_3_^−^, secretion in human airway epithelial cells. We propose that CO_2_‐induced reductions in cytosolic cAMP inhibit CFTR activity and thus CFTR‐dependent Cl^−^ secretion. However, the lack of an effect on pendrin‐dependent HCO_3_^−^ secretion implies that there was sufficient residual CFTR activity to maintain Cl^−^/HCO_3_^−^ exchange by pendrin, and thus efficient HCO_3_^−^ secretion persisted. This is consistent with our previous results in which we showed significant pendrin‐mediated anion exchange activity was still present in Calu‐3 cells where CFTR levels were knocked down by ∼ 75% (Garnett *et al*. 2011). However, dysregulation of CFTR‐dependent Cl^−^ and fluid secretion would be predicted to reduce airways hydration and compromise the innate defence mechanisms of the lungs (Pezzulo *et al*. 2012) predisposing the airways to bacterial colonisation. These findings are of particular relevance to patients suffering from chronic lung diseases, such as chronic obstructive pulmonary disease (COPD) or severe CF, in which bacterial infection is a major problem and hypercapnia is a complication. Thus, based on our findings, hypercapnia may be an additional contributing factor to airways pathophysiology in these situations (Lourenco & Miranda, 1968; Holland *et al*. 2003; Sheikh *et al*. 2011). However, the effects of hypercapnia that we have reported should also be considered for those patients receiving treatment for acute respiratory distress syndrome (ARDS) who suffer from pulmonary oedema due to increased permeability of the alveolar epithelium (Grommes & Soehnlein, 2011). These patients become hypercapnic as a consequence of their clinical treatment (Prin *et al*. 2002) and it has been postulated that it is the elevated CO_2_ that provides the beneficial effects of the treatment. We suggest that a potential protective role of hypercapnia for ARDS patients could be in the reduction in the amount of cAMP‐stimulated fluid secretion in the airways, which would help to minimise the extent of the oedema without compromising the pH‐dependent components of the airway innate defence mechanisms. Interestingly, our findings somewhat contradict those published by the Snzajder group who demonstrated that (i) hypercapnia reduced alveolar fluid reabsorption and thus increased pulmonary oedema in rat alveolar cells (Briva *et al*. 2007; Vadasz *et al*. 2008) and (ii) high CO_2_ increased apical [cAMP]_i_ in both A549 cells and rat alveolar type II cells (Lecuona *et al*. 2013). The findings reported here highlight potential differences in CO_2_ signalling between rat and humans as well as suggest that secretory cells of the conducting airways respond differently to hypercapnia compared to absorptive cells of the respiratory airways. Several studies have also implicated CO_2_ as an anti‐inflammatory agent (Laffey *et al*. 2000; Sinclair *et al*. 2002; De Smet *et al*. 2007; Contreras *et al*. 2012; Oliver *et al*. 2012) whilst hypercapnia has also been shown to attenuate ventilator‐induced lung injury in mice (Otulakowski *et al*. 2014). Our findings may suggest another possible protective role of hypercapnia in ARDS patients which would complement the other reported benefits of hypercapnia.

## Additional information

### Competing interests

None declared.

### Author Contributions

M.J.T., M.J.C. and M.A.G. conceived and designed the experiments. M.J.T., V.S., W.P., S.I. and B.V. conducted experiments and collected data. M.J.T., V.S. and W.P. performed data analysis. J.P.G. and C.W. provided resources. M.J.T., C.W., R.T., M.J.C. and M.A.G. drafted the article or revised it critically for important intellectual content.

### Funding

This work was supported by an MRC Studentship awarded to M.J.T., a BBSRC studentship to W.P. and an overseas studentship to S.H.I. funded by the Higher Committee for Education Development (HCED), Iraq. Additional funding was also provided by the Cystic Fibrosis Trust (Grant SRC003).
